# Assessment of Soybean Flowering and Seed Maturation Time in Different Latitude Regions of Kazakhstan

**DOI:** 10.1371/journal.pone.0166894

**Published:** 2016-12-01

**Authors:** Saule Abugalieva, Svetlana Didorenko, Shynar Anuarbek, Lubov Volkova, Yelena Gerasimova, Ivan Sidorik, Yerlan Turuspekov

**Affiliations:** 1 Institute of Plant Biology and Biotechnology, Almaty, Kazakhstan; 2 Kazakh Research Institute of Agriculture, Almalybak vil., Almaty region, Kazakhstan; 3 East Kazakhstan Research Institute of Agriculture, Solnechnyi vil., Ust-Kamenogorsk region, Kazakhstan; 4 Kostanaiskyi Research Institute of Agriculture, Zarechnoe vil., Kostanai region, Kazakhstan; University of Helsinki, FINLAND

## Abstract

Soybean is still a minor crop in Kazakhstan despite an increase in planting area from 4,500 to 11,400 km^2^ between 2006 and 2014. However, the Government’s recently accepted crop diversification policy projects the expansion of soybean cultivation area to more than 40,000 km^2^ by 2020. The policy is targeting significant expansion of soybean production in South-eastern, Eastern, and Northern regions of Kazakhstan. Successful realization of this policy requires a comprehensive characterization of plant growth parameters to identify optimal genotypes with appropriate adaptive phenotypic traits. In this study 120 soybean accessions from different parts of the World, including 18 accessions from Kazakhstan, were field tested in South-eastern, Eastern, and Northern regions of the country. These studies revealed positive correlation of yield with flowering time in Northern Kazakhstan, with seed maturity time in Eastern Kazakhstan, and with both these growth stages in South-eastern Kazakhstan. It was determined that in South-eastern, Eastern and Northern regions of Kazakhstan the majority of productive genotypes were in maturity groups MGI, MG0, and MG00, respectively. The accessions were genotyped for four major maturity genes (*E1*, *E2*, *E3*, and *E4*) in order to assess the relationship between *E* loci and agronomic traits. The allele composition of the majority of accessions was *e1-as/e2/E3/E4* (specific frequencies 57.5%, 91.6%, 65.0%, and 63.3%, respectively). Accessions with dominant alleles in either *E3* or *E4* genes showed higher yield in all three regions, although the specific genotype associated with greatest productivity was different for each site. Genotype-environment interaction studies based on yield performances suggest that South-east and East regions formed one mega-environment, which was well separated from North Kazakhstan where significantly earlier time to maturation is required. The results provide important insights into the relationship between genetic and phenotypic patterns in new soybean growing territories in Kazakhstan.

## Introduction

Soybean is an important crop globally, with high nutrition, protein and oil values [1], [2]. Despite this it is still a minor crop in Kazakhstan where farmers predominantly grow wheat. However, the recently adopted crop diversification policy in Kazakhstan has driven a steady increase in soybean harvesting area from 4,500 km^2^ in 2006 to 11,400 km^2^ in 2014. In 2012 the Ministry of Agriculture of the Republic of Kazakhstan proposed to increase the area under soybeans to 40,000 km^2^ by 2020, and lands in South-eastern (SEK), Eastern (EK), and Northern (NK) regions of the country were designated for this purpose [3]. Currently, the total yield of soybean has increased from 17,000 tons in 2007 to 20,000 tons in 2014. The increase in production is not straightforward, as yield from 2012 to 2014 did not grow while the sowing area for that period increased nearly to 40,000 hectares [4]. This suggests that in addition to basic agronomic knowledge a detailed understanding of soybean adaptation to the new environments is required.

The productivity of soybean is largely dependent on flowering and maturity times in diverse ecological environments [2], [5], [6], [7]. Therefore, in Northern America 13 maturity groups (MG) were classified for breeding purposes [8]. The classification was found useful as it helped to compare patterns of seed maturation across many different environments [5], [6]. Soybean adaptability is primarily defined by expression of flowering genes [7], which may adjust duration of heading and maturity to maximize resource capture while minimizing the effect of abiotic stresses for specific environments. Flowering genes have been assigned to a series (*E1-E8*) [6], [9]. *E1*, *E2*, *E3*, and *E4* and their roles in flowering time and maturation have been characterized [9], [10], while the identity and function of *E5-E8* genes remains largely unknown [5]. It was shown that *E1-E4* genes are involved in regulation of both pre-flowering and post-flowering growth of plants under different photoperiod length [11]. *E1* is a flowering repressor and encodes a transcription factor that contains a putative nuclear localization signal and region related to the B3 DNA-binding domain [7], [12]. *E2* is an orthologue of the Arabidopsis flowering gene GIGANTEA [2], [13]. *E3* and *E4* encode phytochrome A (PHYA) proteins GmPHYA3 and GMPHYA2, respectively [14], [15]. Dominant alleles of *E1* and *E2*, as well as the *e1-as* allele at the *E1* gene, delay time to maturation, while recessive alleles at *E3* and *E4* provide insensitivity to photoperiod length [11]. It was suggested that in the signaling pathway the two photoreceptor genes *E3* and *E4* suppress transcription of *E1* and correspondingly elevate *GmFT* expression, which directly influences earliness of flowering time [9].

To date in Kazakhstan there are 12 officially registered commercial soybean cultivars basically developed for EK and SEK regions, which have a rather longer history of soybean cultivation than other regions in the country [16]. These cultivars were previously genotyped using DNA microsatellite markers and compared to a large number of foreign accessions [3], [17]. The authors characterized each cultivar with 50 SSR (single sequence repeat) markers and found that local accessions were clearly distinct from those cultivated in Northern America and East Asia [17]. However, no characterization of specific flowering genes has been carried out for these accessions, therefore, little information is available on the genetic bases of soybean adaptation to different environments within Kazakhstan.

The objective of this study was to evaluate whether differences in time of flowering and maturation in a genetically diverse soybean panel grown in different latitudes of Kazakhstan can be associated with particular allele combinations of major *E* series flowering genes. The data obtained provides useful information for selection of the most appropriate parental genotypes to be used in local breeding programs for improved soybean productivity.

## Materials and Methods

**Fig 1:**
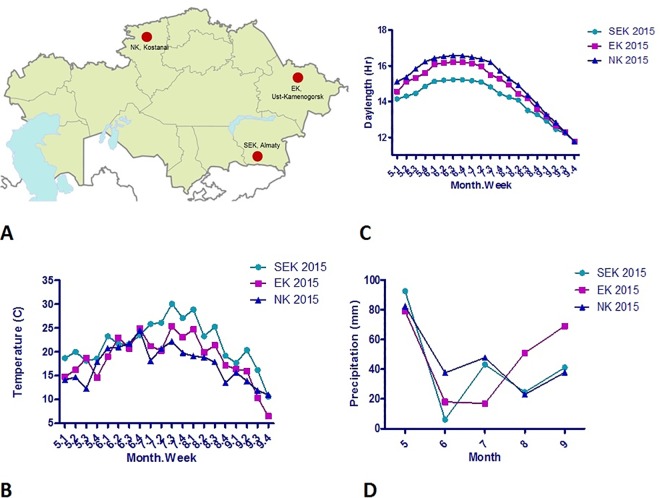
Geographic locations and meteorological data in three experimental sites. (A) The location of the three field trials. (B) The average temperature data by week for the three sites. (C) The average daylength (hours) by week for the three sites. (D) The average precipitation (mm) at the three sites.

The soybean material consisted of 120 genetically diverse cultivars and lines from the collection of the Kazakh Agricultural Research Institute (http://www.kiz.kz, Almaty region, Kazakhstan). The accessions can be obtained by direct request from Dr. Didorenko (svetl_did@mail.ru). The collection is currently a prime source for ongoing breeding projects in SEK, EK, and NK regions of Kazakhstan. The research material represents 12 countries from 5 geographic regions, including Western and Eastern Europe, Northern America, Eastern Asia, and Kazakhstan (S1 Table). It includes 18 accessions from Kazakhstan containing 5 officially registered cultivars in SEK. The collection was grown in three randomized replicates in breeding stations of SEK, EK, and NK regions of Kazakhstan. The locations and meteorological data of field conditions are shown in Fig 1. SEK was irrigated while EK and NK stations were non-irrigated sites. Plants were grown in 1 meter rows with 30 cm between rows and 5 cm between plants within rows. Times to flowering and maturation were studied at R stages described by Fehr et al [18]. The averaged data for triplicated trials were analyzed statistically for assessments of plant growth and yield components in three studied locations. The accessions were genotyped for *E1*, *E2*, *E3*, and *E4* alleles according to protocols published elsewhere [5], [19], [20], [14], respectively. DNA extraction and agarose gel electrophoresis were performed according to Abugalieva et al [17]. Assignment of samples to maturity groups in each region was carried out using thermal time (TT) degrees (^0^Cd) at R8 stage (TT-R8) according to Fehr et al [18]. Time from emergence to flowering was designated as VE-R2, from flowering to pods development as R2-R4, from pods development to seed maturation as R4-R8, and time from emergence to maturation as VE-R8. Statistical analyses of data, including Pearson’s correlation and *t-test*, were calculated by using GraphPad Prism (version 5.00 for Windows, GraphPad Software, San Diego California USA, www.graphpad.com). Genotype-environment interaction patterns, including AMMI (Additive Main Multiplicative Interaction) and GGE Biplot methods, were studied using the GenStat package (16^th^ release, VSN International, Hertfordshire, UK). The symmetric scaling option of both methods and available field data for all three sites were used in estimations.

### Ethics Statement

No specific permissions for field studies were required as all breeding organizations involved are governmental research institutions and participants of the ongoing project supported by the Ministry of Education and Science of the Republic of Kazakhstan.

## Results

### 1. Genetic variation of *E* maturity genes in the soybean collection

One hundred and twenty cultivated soybean accessions grown in Kazakhstan were genotyped for four *E* series genes (S1 Table). The three genotypes found at the highest frequency in the collection were *e1-as/e2/E3/E4* (36 accessions), followed by *e1/e2/E3/e4* (12), and *e1-as/e2/e3/e4* (11). Four alleles were detected for the *E1* gene, where 69 accessions had the *e1-as* allele, 8 accessions had the *e1-fs* allele, 29 accessions had the *e1-nl* allele, and 14 accessions the *E1* allele. The number of recessive dysfunctional alleles were 37 (30.8%), 110 (91.6%), 42 (35%), and 44 (36.7%) for *E1*, *E2*, *E3*, and *E4*, respectively. Separate analysis of the eighteen Kazakh accessions showed different proportions of *E1*, *E3* and *E4* dysfunctional alleles, with 11.1%, 50% and 5.6%, respectively. All 18 samples were fixed for the recessive allele of the *E2* gene (S1 Table). Nine out of eighteen local accessions had the *e1-as/e2/E3/E4* genotype.

Five major groups of ten or more accessions were found among the seventeen *E* genotype combinations found in this study (Table 1). The number of genotypes characterised was reduced to sixteen in EK and fifteen in NK because accessions with genotypes *e1-n/E2/E3/E4* in NK and *E1/e2/E3/E4* in EK and NK did not form seeds.

**Table 1:** Yield performance of seventeen genotypes of soybean at three experimental sites.

Genotypes	SEK 2015 (N 43°15′, E 76°54’)			EK 2015 (N 49°57′, E 82°37’)			NK 2015 (N 53°12′, E 63°37’)		
	n	MG	Yield per plant (g)	N	MG	Yield per plant (g)	n	MG	Yield per plant (g)
*E1/E2/e3/E4*	2	00/0	17.8±1.5	2	000/00	25.4±1.1	2	000	12.0±2.9
*E1/e2/E3/E4*	1	I	30.3±0.0	n/a	n/a	n/a	n/a	n/a	n/a
*E1/e2/E3/e4*	7	00/0	13.0±2.5	7	00	21.3±3.6	6	000/00	40.9±10.2
*E1/e2/e3/E4*	3	0/I	23.4±3.7	2	000/00	31.9±10.6	2	00	18.4±6.7
*E1/e2/e3/e4*	1	0	14.6±0.0	1	00	23.6±0.06	1	00	24.4±0.0
*e1-as/E2/E3/E4*	5	00	15.8±2.3	5	000/00	24.0±2.3	4	000/00	25.2±2.8
*e1-as/e2/E3/E4*	36	000/00/0/I/II	17.6±1.2	35	000/00/0	24.8±1.5	26	000/00	23.2±1.8
*e1-as/e2/E3/e4*	6	000/00/0	14.1±2.5	6	000/00	29.4±4.2	3	000/00	35.7±4.5
*e1-as/E2/e3/E4*	1	00	19.9±0.0	1	00	31.3±0.0	1	000	39.1±0.0
*e1-as/e2/e3/E4*	10	00/0/I	19.4±1.9	9	000/00/0	20.1±2.5	7	000/00	21.7±2.9
*e1-as/e2/e3/e4*	11	00/0	17.5±1.8	11	000/00	23.6±2.9	8	000/00	23.2±4.7
*e1-n/E2/E3/E4*	1	II	18.3±0.0	1	0	62.5±0.0	n/a	n/a	n/a
*e1-n/e2/E3/E4*	10	00/0/I	17.7±1.8	10	000/00/0	34.0±7.1	6	000/00	23.8±2.4
*e1-n/e2/E3/e4*	12	000/00/0/I	16.0±1.5	12	000/00/0	25.6±3.3	9	000/00	22.6±4.3
*e1-n/E2/e3/E4*	1	I	18.9±0.0	1	00	18.5±0.0	1	000	13.8±0.0
*e1-n/e2/e3/E4*	6	00/0/I	19.2±1.5	6	000/00	30.0±3.1	4	000/00	16.1±5.3
*e1-n/e2/e3/e4*	7	00	12.3±1.7	7	000/00	17.3±1.8	5	000/00	23.6±3.7

SEK, South-eastern Kazakhstan; EK, Eastern Kazakhstan; NK, Northern Kazakhstan; n, number of accessions; MG, maturity group.

### 2. Evaluation of adaptability of soybean collection in three regions of Kazakhstan

The diverse collection (S1 Table) was analyzed for key plant growth stages by three breeding organizations representing SEK, EK and NK. Accessions were studied for the length of six developmental stages at TT-R2 and TT-R8 stages (S2 Table).

As locations differed in latitude and longitude there were differences in daylength, meteorological conditions (Fig 1), and sowing dates (S2 Table). The correlations for all growth stages across the three locations was very high (S3 Table), except for stage R4-R8 where the correlation index between SEK and NK stations was less significant (P <0.02). The TT-R2 duration was similar between SEK and EK sites, which was 300 degrees shorter in NK (Table 2). The duration of TT-R8 in SEK was longer by nearly 300 degrees compared to EK and NK sites, indicating that the greatest contrast at these two stages is between SEK and NK (Table 2).

**Table 2:** Average data from triplicated experiments on plant growth stages and yield parameters in three studied sites.

	SEK 2015 (n = 120)	EK 2015 (n = 115)	NK 2015 (n = 85)
VE-R2 (d)	32.09±0.284	27.30±0.461	44.86±0.675
TT-R2 (^0^Cd)	645.9±6.105	643.9±8.869	955.2±14.47
R2-R8 (d)	65.25±0.746	65.04±1.023	61.41±0.857
R4-R8 (d)	38.06±0.45	49.37±0.82	44.56±0.62
VE-R8 (d)	97.27±0.873	98.14±1.138	106.3±1.137
TT-R8(^0^Cd)	2314±18.6	2060±18.71	2013±13.85
PH (cm)	63.90±1.881	63.97±1.141	81.59±1.792
NFB (n)	1.758±0.069	2.791±0.095	4.865±0.273
NPP(n)	18.64±0.470	30.64±0.941	37.38±1.743
NSP(n)	51.96±1.369	74.70±2.431	78.18±4.406
YPP (g)	17.09±0.577	25.93±1.288	24.22±1.314
TSW (g)	175.4±4.614	159.8±2.122	155.1±2.397

SEK, South-east Kazakhstan; EK, East Kazakhstan; NK, North Kazakhstan; VE-R2, from emergence to flowering (days); TT-R2, thermal time length at flowering (^0^Cd); R2-R8, from flowering to maturity (days); R4-R8, from pods development to maturity (days); VE-R8, from emergence to maturity (days); TT-R8, thermal time length at maturity (^0^Cd); PH, plant height (cm); NFB, number of fertile branches; NPP, number of pods per plant; NSP, number of seeds per plant; YPP, yield per plant (g); TSW, thousand seeds weight (g).

The durations of TT-R8 were used to classify accessions into maturity groups (MG) in the three sites (Table 3). Since the collection was characterized by 5 MGs in SEK, 3 MGs in EK, and only 2 MGs in NK, it was concluded that the number of MGs reduced in higher latitudes. The largest groups were MG00 in SEK and EK (86 and 61 accessions, respectively), and MG000 (44) in NK. Local breeding lines fell into the largest groups, except in SEK where Kazakh accessions were assigned to MGI and MGII (S1 Table). In each site, the highest average yield per plant (YPP) was recorded for the latest maturity group (Table 3).

**Table 3:** Number of accessions and average yield in maturity groups at three studied sites.

Sites	∑ n	MG000		MG00		MG0		MGI		MGII	
		n	YPP	n	YPP	n	YPP	n	YPP	n	YPP
SEK	120	3	9.9±1.0	86	15.2±0.6	13	19.8±0.9	16	24.9±1.1	2	27.6±9.3
EK	115	44	19.9±0.9	61	27.7±1.5	10	36.0±4.6	n/a	n/a	n/a	n/a
NK	85	44	21.3±1.5	41	27.4±2.1	n/a	n/a	n/a	n/a	n/a	n/a

SEK, South-eastern Kazakhstan; EK, Eastern Kazakhstan; NK, Northern Kazakhstan; n, number of accessions; MG, maturity groups; YPP, yield per plant (g); n/a, not available.

#### Performance of soybean collection in South-eastern region

Four out of five local cultivars belonged to MGI, which contained 16 accessions. The fifth local accession was one of the two samples in MGII (S1 Table). As the TT-R8 duration of the 16 samples in MGI was significantly higher than those of MG0 and MG00 accessions (P<0.0001), their yield components were assessed in comparison to samples from two earlier flowering MGs. It was also determined that MGI was superior to MG0 and MG00 for the average YPP (Table 3). The highest yielding individual accessions were SD74 and SD117, which were characterized by the *e1-as/e2/E3/E4* genotype and assigned to MGI and MGII groups, respectively (S1 Table). It was noted that the local standard cultivar SD116 (*E1/e2/E3/E4*) also performed as a high yield accession with late time to flowering and maturation.

The role of each gene in plant performance was examined by comparing field data for each parameter. For the SEK location a two-tailed t-test indicated that alleles at *E1* and *E4* were highly significant for plant growth performance (S4 Table). Specifically, diversity at *E1* was statistically significant for time to flowering (P<0.001), while that at *E4* played an important role for time to maturity and yield (P<0.01). In pairwise analysis significant allele combinations for maturation and YPP were also recorded for *E1/E4* (P<0.001). This trend was confirmed for three *E* gene comparisons (S4 Table).

#### Performance of soybean collection in Eastern region

Five local accessions bred in SEK did not form pods in the EK field conditions and, therefore, only 115 out of 120 accessions were analyzed. The TT-R8 values of the collection fell into 3 MGs (Table 3) suggesting that later ripening groups may not be suited to the region. The analysis of growth stages showed similar TT-R2 duration and 264 degree shorter TT-R8 duration in comparison with the SEK region. Most accessions (61) of the collection were grouped into MG00, including 4 out of 5 accessions bred in EK region. However, the highest YPP was recorded in the smallest group MG0 (10 accessions). Within MG0 three accessions had *e1-as/e2/e3/E4*, three accessions *e1-as/e2/E3/E4*, two accessions *e1/e2/E3/E4*, and the remaining two accessions had *e1/e2/E3/e4* and *e1/E2/E3/E4* genotypes. SD91 (*e1/e2/E3/e4*) and SD82 (*e1/e2/E3/E4*) showed 31 days to flowering time and were the earliest flowering genotypes within MG0. The latest flowering accession was SD75 (*e1-as/e2/e3/E4*), followed by SD74 (*e1-as/e2/E3/E4*), which flowered at 49.7 and 49.0 days, respectively. When averages of flowering time for these contrasting pairs of accessions were compared by using yield data, it was determined that early flowering genotypes were maturing 13.5 days later and their yield per plant was higher by 11.4 g.

Nine accessions marked in S1 Table were also analyzed in a high latitude region of China [6]. Therefore, it was interesting to compare MG reference varieties with results in this study (Table 4). The data from field trials in EK was selected for comparison, as latitude in this site was similar to reported Chinese conditions (49^0^57’ vs 50^0^15’). It appeared that 5 out of 9 accessions showed similar MGs, while 4 others were found in earlier MGs in China.

**Table 4:** Comparative maturity groups in reference soybean varieties at two different studies.

Soybean accessions			This study			Zia et al., 2014
ID	Varieties	Origin	MG SEK	MG EK	MG NK	China
SD003	ОАС Vision	Canada	00	00	na	000
SD004	Maple Presto	Canada	00	000	000	000
SD008	Maple Ridge	Canada	00	00	na	00
SD017	Heyhek 14	China	00	00	000	000
SD034	Lydia	Russia (Far-East)	00	00	na	00
SD051	Sonata	Russia (Far-East)	00	00	na	000
SD052	Sunset	Russia (Far-East)	00	000	000	0000
SD060	Harmony	Russia (Far-East)	00	00	00	00
SD080	Terek	Ukraine	I	00	na	00

SEK, South-eastern Kazakhstan; EK, Eastern Kazakhstan; NK, Northern Kazakhstan; MG, maturity groups.

The *t-test* was applied in order to determine the significance of each gene and their combinations in the analysis of growth stages and yield components for all studied 115 accessions in EK (S4 Table). It was found that the *E1* genotype is statistically significant for pre-flowering time (P<0.001), and *E4* for post-flowering time (P<0.05). The analysis of combinations of alleles for three genes (*E1*, *E3*, *and E4*) allowed the identification of 11 different allelic combinations with the largest group being 39 *e1-as/E3/E4* accessions. However, similar to the MG studies, the most productive genotype was found to be *e1/E3/E4* (11 accessions). In the study of 115 accessions, *e1/E3/E4* showed earlier flowering (3.4 days in average) and later maturation time (10.3 days in average) than *e1-as/E3/E4*. In accessions with an *e1* background all four combinations of *E3* and *E4* alleles showed similar flowering time (30.5–30.9 days) but different time to maturation. The earliest maturation (R2-R8) was recorded for *e3/e4* (57.5 days) followed by *E3/e4* (65.5), *e3/E4* (71.3), and *E3/E4* (74.0). Hence the largest difference between these groups was 16.5 days, which was the biggest factor contributing to the YPP in the region (P<0.0001). It is interesting to note that none of five local promising breeding lines was found to have the *e1/E3/E4* genotype in EK (S1 Table).

#### Performance of soybean collection in Northern region

NK was the highest latitude testing site in this work and only 85 accessions of the collection formed seeds in all three replication blocks. Therefore, only those 85 accessions were selected for further comparative studies. The YPP in NK and EK were mostly similar to each other, but significantly higher than in SEK (P<0.0001, Table 2). The TT-R8 values in NK separated the accessions into MG00 and MG000, with the number of accessions nearly evenly split between the two groups (41 and 44, respectively; Table 3). All eight local accessions from NK were grouped into MG000, although the TT-R8 was longer (P<0.0001) and the YPP was higher (P<0.05) in MG00 in comparison to MG000. Within MG00 the largest group was *e1-as/e2/E3/E4* genotype (14 accessions) and the highest YPP was recorded for the *E1/e2/E3/e4* genotype (5 accessions), which included three accessions from the Ukraine (S1 Table).

Pairwise comparisons of genes showed that *E1*and *E4* showed the highest statistical correlation with time to flowering and maturity and YPP (S4 Table). As in the other two environments (SEK and EK), the individual contributions of *E2* alleles to flowering and maturation in NK were not significant in this study. In three gene analyses the most frequent allelic combination was *e1-as/E3/E4* (30 accessions), which included five locally bred accessions. In an *E1* background all three genotypes had the same duration of seed maturation but differed by 4.2 days in flowering time between *E3/e4* and *e3/E4* genotypes, suggesting that late flowering is beneficial for higher yield components in the region. The comparison of *E1/E3/e4* and *e1-as/E3/E4* using *t-test* showed statistically significant difference for duration of R2 and R8, and YPP (P<0.01).

### 3. Genotype-environment interaction patterns

Flowering and seed maturation times, as two key developmental phases of soybean, were analyzed for correlation to YPP in the three sites (S2 Table). Late flowering time was positively correlated with yield in SEK (P<0.0001) and NK (P<0.001) but not in EK. Late maturation time was very significant for the yield in SEK and EK (P<0.0001, for both cases), but was insignificant for NK site. The comparison of time to flowering and maturity in top productive genotypes in each region to performances of genotypes recommended for each MG reported in [2] is shown in Table 5. In SEK and EK regions the two genotypes with highest productivity in either site have been selected because their best genotype was represented by one accession only (Table 5). Results confirmed the advantages of the selected top genotypes (Table 1) and suggested the optimal range of time to flowering and maturity for each experimental site. The allele combinations in four *E* genes between selected top genotypes in this study and suggested genotypes for each MG proposed in [2] were different in all three sites (Table 5). There were differences in growth performances of top high-yielding genotypes among studied sites, indicating the reduction of TT-R8 duration from 2754 ^0^Cd in SEK to 2138 ^0^Cd in NK.

**Table 5:** Comparison of ranges of time to flowering and maturation in genotypes recommended for different latitude regions.

Regions	MG	Genotypes	n	TT-R2 (^0^Cd)	TT-R8 (^0^Cd)	Yield per plant (g)
SEK	MGI	*E1/e2/E3/E4*	1	730.5±00.0	2754±00.0	30.31±0.0
SEK	MG0/MGI	*E1/e2/e3/E4*	3	764.0±40.8	2606±85.5	23.43±3.7
SEK	MG0*/MGI*	e1-as/E2/e3/E4*	1	705.7±0.00	2375±0.00	19.90±0.0
SEK	MG0*/MGI*	e1-as/e2/e3/E4*	10	655.0±24.1	2380±60.5	19.38±1.2
SEK	MG0*/MGI*	e1-as/e2/E3/E4*	36	648.5±11.7	2301±34.0	17.56±1.2
EK	MG0	*e1-n/E2/ E3/E4*	1	670.0±00.0	2418±00.0	62.51±0.0
EK	MG000/MG00/ MG0	*e1-n/e2/ E3/E4*	10	576.9±11.8	2123±83.2	33.99±7.1
EK	MG00*/MG0*	e1-as/e2/E3/E4*	34	669.1±17.7	2073±32.3	24.75±1.5
EK	MG00*/MG0*	e1-as/e2/e3/E4*	9	676.8±44.0	2102±91.2	20.08±2.5
EK	MG000*	e1-n/e2/e3/e4*	7	586.3±10.3	1889±35.7	17.34±1.8
NK	MG00	*E1/e2/E3/e4*	6	1115±28.7	2138±33.2	40.93±10.2
NK	MG00*	e1-as/e2/E3/E4*	26	957.7±27.9	2004±21.6	23.16±1.8
NK	MG00*	e1-as/e2/e3/E4*	7	941.2±60.9	2031±59.3	21.69±2.9

SEK, South-eastern Kazakhstan; EK, Eastern Kazakhstan; NK, Northern Kazakhstan; MG, maturity groups; TT-R2, thermal time length at flowering (^0^Cd); TT-R8, thermal time length at maturity (^0^Cd); bold*, proposed genotypes for different maturity groups according to [2].

**Fig 2:**
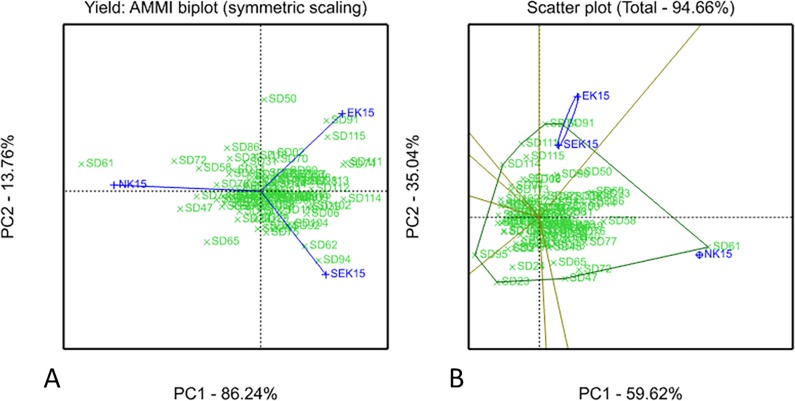
**AMMI (A) and Biplot (B) graphics on genotype–environment interaction studies.** Analyses based on yield data of soybean collection grown in South-east Kazakhstan (SEK15), East-Kazakhstan (EK15), and Northern Kazakhstan (NK15) harvested in 2015. PC1 and PC2 are Principal Coordinates of the analyses. Environment and genotype scores are given in blue and green colors, respectively.

The YPP values from field trials were used for estimation of genotype-environment interaction (GEI) patterns based on AMMI and GGE Biplot methods (Fig 2). The AMMI effects of ANOVA (analysis of variance) detected large environmental contribution to the interaction (89.52%), while the sum of Genotype (G) and Genotype x Environment (GE) impacts was only 10.48%. The principal coordinate analysis of AMMI (Fig 2A) suggested that PC1 effectively discriminated SEK and EK sites from NK site, and PC2 allowed the differentiation of SEK from EK. Rather similar results were obtained using GGE Biplot methods, although in this case SEK and EK were combined to the same Mega-environment (Fig 2B).

## Discussion

### 1. Characterization of genotyping diversity in tested soybean accessions

Despite soybean being a typical short-day species it is successfully cultivated in long-day high latitude regions of North America, Europe and China [2], [6]. There are many reports suggesting that adaptation to certain environments is directly related to the relationship between flowering gene alleles and time to maturation of soybean [7], [21], [22]. Hence, it was important to find out which genotypes show highest productivity in different regions of Kazakhstan. Therefore, the breeding collection of soybean both in current harvesting areas and in recently developed high latitude territories in Kazakhstan was characterized for *E* flowering gene composition. Five major groups of genotype combinations (n >10) were identified from the seventeen variants found for the four *E* gene alleles of the breeding collection in this study (Table 1). The genotypic structure of the breeding collection was comparable to those reported in China [2], [6] and for photoperiod-insensitive accessions in Japan [11]. It was found that most accessions tested in Chinese reports were *E1/e2/E3/E4* followed by *e1-as/e2/e3/E4*, while in the Japanese report they were *e1-as/e2/e3/e4* followed by *e1-as/e2/e3/E4* and *e1/e2/e3/E4*. The largest group of accessions in the current study was *e1-as/e2/E3/E4*, which was moderately common in studies in China but absent in field trials in Japan. The discrepancy in genotypic content in this study may be due to more frequent exchange of germplasm with partners from those Eastern European countries that were part of former USSR.

### 2. Comparison of MG in regions with different latitudes

The comparison of MGs in three different latitude regions of Kazakhstan showed a reduction from five groups in SEK (MGII-MG000) to 2 groups in NK (MG00-MG000); in each trial the latest MG always provided better yield (Table 3). In 23 out of 120 soybean accessions the MG groups were similar across all three sites, and 93 accessions showed similarity for at least two sites, therefore, it is still a convenient instrument for breeding purposes [2, 5]. In spite of a few exceptions the trend was the reduction of TT-R8 duration from warmer to colder regions with higher latitude (Table 3). Since the highest yield components were registered for EK and NK (Table 2) it was important to determine an optimum duration of growth in those previously unstudied high latitude regions. It was found that the most productive accessions in EK and NK have the averages 2150 and 2138 for TT-R8, respectively (Table 5). Therefore, the accessions within this range of maturation were assigned to MG00. According to [2], MG0 and MG00 both were genotyped as *e1-as/e2/E3/E4* and *e1-as/e2/e3/E4*. However, in the study the most optimal MG00 genotypes were *e1-n/e2/E3/E4* and *e1-n/E2/E3/E4* in EK, and *E1/e2/E3/e4-n* in NK, suggesting that additional genetic factors associated with time to maturation should be involved in the determination of maturity groups.

### 3. Associations of flowering genes and maturity time in different latitude regions

The *E* series of genes were shown to be important factors for plant adaptation in soybean growing regions [12], [14]. It was recently hypothesized that three of those *E* genes (*E1*, *E3*, *and E4*) play larger roles in pre- and post-flowering photoperiod responses [11]. The authors underlined the role of *E3* and *E4* genes in photoperiod insensitive genotypes for early flowering and seed maturation. In particular, they proposed a gene regulatory network comprising three known maturity genes, a determinate habit gene (*Dt1*) and two unknown factors (X, Y) that regulate flowering under long day conditions. Results in this study suggests that alleles of *E1* and *E4* and their interaction, with a contribution from *E1/E3/E4* interactions, are most important in prediction of flowering time in different latitude regions. The frequency of the *E2* dominant allele in this study was small and the effect of the gene was statistically insignificant for plant growth parameters in all tested regions. In the SEK region the single accession of *E1/e2/E3/E4* (SD116, local commercial cultivar “Zara”) had the latest pre- and post-flowering and the highest yield in comparison with averages of sixteen other genotypes, including *e1-as/e2/E3/E4* and *e1/e2/E3/E4*. The duration of VE-R8 stage in *E1/e2/E3/E4* was lower than in *e1-as/e2/E3/E4* and *e1/e2/E3/E4* genotypes for 22.0 and 17.2 days, respectively. Analysis of time to flowering and maturation suggested that SEK was the only studied region where VE-R2 and R2-R8 have significant correlation (P<0.001, S3 Table).

In higher latitude regions of EK and NK the patterns of associations between flowering genes and yield were different to some extent. The highest yield in the collection in EK was recorded for the *e1/e2/E3/E4* genotype (11 accessions). Dominant alleles of *E3/E4* significantly delayed the R2-R8 stage resulting in the longest VE-R8 among 17 genotypes (S5 Table). The *e1/e2/E3/E4* genotype showed 3.4 days earlier flowering and 10.3 days later post-flowering difference in comparison with *e1-as/e2E3/E4* suggesting that *E3/E4* is prolonging seed maturation when *E1* and *E2* are dysfunctional. The GEI analysis based on yield performance suggested that early flowering time is essential in SEK and EK, and, therefore, this result effectively separated SEK and EK from NK (Fig 2).

## Conclusions

This study is the first attempt to assess soybean adaptation in different latitude regions of Kazakhstan based on *E* gene variation and field trial results. Although *e1-as/e2/E3/E4* was the most common genotype in this collection, it was not among the top yield performing genotypes in three studied regions. Specific allele combinations of the four *E* genes and optimal ranges of time to flowering and maturity were proposed for each experimental site based on identification of top genotypes with higher productivity. The results confirmed the high importance of *E1* for length of flowering, and of *E3/E4* for time to maturation. The MG analyses based on yield performance and GGE Biplot method showed that SEK and EK regions have more similarity and comprised one mega-environment while NK region formed another.

## Supporting Information

S1 Table The list of soybean accessions studied for four *E* genes and maturity groups in three regions of Kazakhstan. (DOC)

S2 Table Phenotypic and agronomic data from three experimental sites of Kazakhstan. SEK2015, EK2015, NK2015 –averaged data from 2015 harvest in South-east Kazakhstan, East Kazakhstan, and North Kazakhstan, respectively.
(XLS)

S3 Table Correlation coefficient values in three experimental sites among plant growth stages and yield parameters. (XLS)

S4 Table Relationships between *E* gene combinations and TT-R2, TT-R8 length and yield based on two-tailed t-test. SEK, EK, and NK are South-east, East, and North regions in Kazakhstan, respectively. *—P<0.05; **—P<0.01; ***—P<0.001; ****—P<0.0001; YPP–yield per plant.
(XLS)

S5 Table Averages of field data of soybean collection for genotypes of three *E* genes (*E1*, *E3*, and E4) at three experimental sites. (XLS)

## References

[1] Duranti M. Grain legume proteins and nutraceutical properties Fitoterapia; 2006;77:67–82. 10.1016/j.fitote.2005.11.008 16406359

[2] Jiang B, Nan H, Gao Y, Tang L, Yue Y, Lu S, et al Allelic Combinations of Soybean Maturity Loci E1, E2, E3 and E4 Result in Diversity of Maturity and Adaptation to Different Latitudes. PLoS ONE; 2014;9(8): e106042 10.1371/journal.pone.0106042 25162675 PMC4146597

[3] Anuarbek S, Volkova L, Turuspekov Y, Abugalieva S. Screening of soybean world collection using DNA markers. KazNU Bulletin. Biology series; 2015;3(65):110–17. Russian.

[4] FAOSTAT (FAO), [Internet]. Rome, Italy. [Accessed 2016 March 12]. Available from http://faostat3.fao.org/home/E

[5] Zhai H, Lü S, Wang Y, Chen X, Ren H, Yang J, et al Allelic Variations at Four Major Maturity E Genes and Transcriptional Abundance of the E1 Gene Are Associated with Flowering Time and Maturity of Soybean Cultivars. PLoS ONE; 2014;9(5), e97636 10.1371/journal.pone.0097636 24830458 PMC4022622

[6] Jia H, Jiang B, Wu C, Lu W, Hou W, Sun S, et al Maturity Group Classification and Maturity Locus Genotyping of Early-Maturing Soybean Varieties from High-Latitude Cold Regions. PLoS ONE; 2014; 9(4): e94139 10.1371/journal.pone.0094139 24740097 PMC3989213

[7] Watanabe S, Harada K, Abe J. Genetic and molecular bases of photoperiod responses of flowering in soybean. Breeding Science; 2012;61:531–43. 10.1270/jsbbs.61.531 23136492 PMC3406791

[8] Zhang L, Kyei-Boahen S, Zhang J, Zhang M, Freeland T, Watson C, et al Modifications of optimum adaptation zones for soybean maturity groups in the USA. Crop Management; 2007;6(1).

[9] Tsubokura Y, Watanabe S, Xia Z, Kanamori H, Yamagata, Kaga A, et al Natural variation in the genes responsible for maturity loci E1, E2, E3 and E4 in soybean. Annals of Botany; 2014;113:429–41 10.1093/aob/mct269 24284817 PMC3906962

[10] Xia Z, Zhai H, Liu B, Kong F, Yuan X, Wu H, et al Molecular identification of genes controlling flowering time, maturity, and photoperiod response in soybean. Plant Syst Evol; 2012;298:1217–27.

[11] Xu M, Xu Z, Liu B, Kong FJ, Tsubokura Y, Watanabe S, et al Genetic variation in four maturity genes affects photoperiod insensitivity and PHYA-regulated postflowering responses of soybean. BMC Plant Biology; 2013;13:91–04. 10.1186/1471-2229-13-91 23799885 PMC3698206

[12] Xu M, Yamagishi N, Zhao C, Takeshima R, Kasai M, Watanabe S, et al The Soybean-Specific Maturity Gene E1 Family of Floral Repressors Controls Night-Break Responses through Down-Regulation of FLOWERING LOCUS T Orthologs. Plant Physiology; 2015;168:1735–46. 10.1104/pp.15.00763 26134161 PMC4528769

[13] Watanabe S, Xia Z, Hideshima R, Tsubokura Y, Sato S, Yamanaka N, et al A map-based cloning strategy employing a residual heterozygous line reveals that the GIGANTEA gene is involved in soybean maturity and flowering. Genetics; 2011;188:395–07. 10.1534/genetics.110.125062 21406680 PMC3122305

[14] Liu B, Kanazawa A, Matsumura H, Takahashi R, Harada K, Abe J. Genetic redundancy in soybean photoresponses associated with duplication of phytochrome A gene. Genetics; 2008;180:996–07. 10.1534/genetics.108.092742 PMC2567397 18780733

[15] Watanabe S, Hideshima R, Xia Z, Tsubokura Y, Sato S, Nakamoto Y, et al Map-based cloning of the gene associated with the soybean maturity locus E3. Genetics; 2009;182:1251–62. 10.1534/genetics.108.098772 19474204 PMC2728863

[16] Abugalieva S. Genetic diversity of soybean (*Glycine max* (L.) Merrill). Biotechnology. Theory and Practice; 2013;4:13–9. Russian.

[17] Abugalieva S, Volkova L, Nurlanova A, Zhanpeisova A, Turuspekov Y. DNA fingerprinting of soybean cultivars from Kazakhstan by using microsatellite markers. Biotechnology. Theory and Practice; 2013;3:26–34.

[18] Fehr W, Caviness C, Burmood D, Pennington J. Stage of development descriptions for soybeans, Glycine max (L.) Merrill. Crop Science; 1971;11:929–31.

[19] Shin JH, Lee SH. Molecular markers for the E2 and E3 genes controlling flowering and maturity in soybean. Molecular Breeding; 2012;30:1793–8.

[20] Liu B, Watanabe S, Uchiyama T, Kong F, Kanazawa A, Xia Z, et al The soybean stem growth habit gene *Dt1* is an ortholog of Arabidopsis TERMINAL FLOWER 1. Plant Physiology; 2010;153:198–10. 10.1104/pp.109.150607 20219831 PMC2862436

[21] Buzzell RI, Voldeng HD. Inheritance of insensitivity to long day length. Soybean Genetic Newsletters; 1980;7:26–9.

[22] Saindon G, Voldeng HD, Beversdorf WD, Buzzell RI. Genetic control of long daylength response in soybean. Crop Science; 1989;29:1436–9.

