# Supplementary material for: Assessment of Soybean Flowering and Seed Maturation Time in Different Latitude Regions of Kazakhstan
Source: PLoS One. 2016 Dec 1;11(12):e0166894. doi: 10.1371/journal.pone.0166894 (PMC5132232; doi:10.1371/journal.pone.0166894)
Supplement: S1 Table — (DOC) [file pone.0166894.s001.doc]

| **S1 Table. The list of soybean accessions studied for four E genes and maturity groups in three regions of Kazakhstan.** | | | | | | | |
| --- | --- | --- | --- | --- | --- | --- | --- |
| **Catalogue ID** | **Sample ID** | **Origin** | **E series loci genotype** | **MG SEK15** | **MG EK15** | **MG NK15** |  |
| SD01 | Yaselda | Belarus | *e1-nl/e2/E3/e4* | 0 | 00 | 00 |  |
| SD02 | Pripyat | Belarus | *e1-nl/e2/E3/e4* | 00 | 00 | 00 |  |
| SD03* | ОАС Vision | Canada | *e1-nl/e2/E3/e4* | 00 | 00 | na |  |
| SD04* | Maplepresto | Canada | *e1-nl/e2/e3/e4* | 00 | 000 | 000 |  |
| SD05 | Emerson | Canada | *e1-nl/e2/E3/e4* | 0 | 00 | 00 |  |
| SD06 | P-73-3 | Canada | *e1-as/e2/E3/E4* | 00 | 00 | 00 |  |
| SD07 | Supra | Canada | *e1-nl/e2/E3/E4* | I | 0 | na |  |
| SD08* | Maple Ridge | Canada | *e1-nl/e2/e3/E4* | 00 | 00 | na |  |
| SD09 | Maplearrow | Canada | *e1-nl/e2/E3/E4* | I | 00 | na |  |
| SD10 | Mapleglen | Canada | *e1-nl/e2/E3/E4* | I | 00 | na |  |
| SD11 | Mapleamber | Canada | *e1-nl/e2/E3/E4* | 00 | 00 | 000 |  |
| SD12 | Gaillard | Canada | *e1-nl/e2/E3/E4* | 00 | 00 | 00 |  |
| SD13 | KG 20 | Canada | *e1-as/e2/e3/e4* | 00 | 00 | na |  |
| SD14 | Accord | Canada | *e1-as/E2/e3/E4* | 00 | 00 | 000 |  |
| SD15 | АС Brant | Canada | *e1-as/e2/E3/E4* | 0 | 00 | na |  |
| SD16 | Harbin | China | *e1-as/e2/e3/E4* | 0 | 0 | na |  |
| SD17* | Heyhek 14 | China | *e1-as/e2/E3/E4* | 00 | 00 | 000 |  |
| SD18 | LMF | Poland | *e1-nl/e2/E3/E4* | 00 | 00 | 000 |  |
| SD19 | Chabem Wekoju | Poland | *e1-as/e2/E3/E4* | 00 | 00 | 000 |  |
| SD20 | Arctic | Poland | *e1-nl/e2/e3/e4* | 00 | 000 | na |  |
| SD21 | Kollekcyina | Poland | *e1-nl/e2/E3/e4* | 000 | 000 | 000 |  |
| SD22 | Nawiko | Poland | *e1-nl/e2/E3/e4* | 00 | 000 | na |  |
| SD23 | Warsawska | Poland | *E1/e2/E3/e4* | 00 | 00 | 000 |  |
| SD24 | Kasatka | Russia | *e1-as/e2/E3/E4* | 00 | 000 | 000 |  |
| SD25 | Severnaya 5 | Russia | *e1-nl/e2/e3/e4* | 00 | 000 | 000 |  |
| SD26 | Smena | Russia | *e1-as/e2/E3/E4* | 00 | 000 | 00 |  |
| SD27 | Rassvet | Russia | *e1-as/e2/E3/E4* | 00 | 00 | 00 |  |
| SD28 | Amurskaya 401 | Russia | *e1-nl/e2/E3/e4* | 00 | 000 | 000 |  |
| SD29 | Soer 3491 | Russia | *e1-as/e2/E3/E4* | 00 | 00 | 00 |  |
| SD30 | Omskaya 4 | Russia | *e1-as/e2/E3/E4* | 00 | 000 | 000 |  |
| SD31 | Soer 3 | Russia | *e1-as/e2/E3/E4* | 00 | 00 | 00 |  |
| SD32 | Bryanskaya | Russia | *e1-as/e2/E3/E4* | 00 | 00 | 00 |  |
| SD33 | Nadezhda | Russia | *e1-as/e2/e3/e4* | 00 | 00 | 00 |  |
| SD34* | Lidiya | Russia | *e1-nl/e2/e3/E4* | 00 | 00 | na |  |
| SD35 | VNIIS-1 | Russia | *e1-as/e2/E3/E4* | 00 | 00 | na |  |
| SD36 | Luchezarnaya | Russia | *e1-as/E2/E3/E4* | 00 | 00 | 00 |  |
| SD37 | PEP 27 | Russia | *e1-nl/e2/E3/e4* | 00 | 000 | na |  |
| SD38 | Sibniik 315 | Russia | *e1-nl/e2/E3/e4* | 00 | 000 | 000 |  |
| SD39 | VNIIS-2 | Russia | *e1-as/e2/E3/E4* | 00 | 00 | 000 |  |
| SD40 | Soer 4 | Russia | *e1-as/e2/e3/E4* | 00 | 00 | 00 |  |
| SD41 | Bara | Russia | *e1-as/e2/e3/e4* | 00 | 00 | na |  |
| SD42 | Zolotistaya | Russia | *e1-as/e2/E3/E4* | 00 | 000 | na |  |
| SD43 | Zlata | Russia | *e1-as/e2/e3/e4* | 00 | 00 | 000 |  |
| SD44 | Mageva | Russia | *e1-as/E2/E3/E4* | 00 | 000 | 000 |  |
| SD45 | Soer 5 | Russia | *e1-as/e2/E3/E4* | 000 | 000 | 000 |  |
| SD46 | Okskaya | Russia | *e1-as/E2/E3/E4* | 00 | 000 | 000 |  |
| SD47 | Svetlaya | Russia | *e1-as/e2/E3/e4* | 000 | 000 | 000 |  |
| SD48 | Maleta | Russia | *e1-nl/e2/e3/e4* | 00 | 000 | 000 |  |
| SD49 | Vega | Russia | *e1-as/e2/E3/E4* | I | 00 | 00 |  |
| SD50 | Luch Nadezhdy | Russia | *e1-as/e2/e3/e4* | 00 | 00 | na |  |
| SD51* | Sonata | Russia | *e1-as/e2/E3/E4* | 00 | 00 | na |  |
| SD52* | Zakat | Russia | *e1-as/E2/E3/E4* | 00 | 000 | 000 |  |
| SD53 | Svapa | Russia | *e1-fs/e2/E3/E4* | 00 | 000 | 000 |  |
| SD54 | Lantsetnaya | Russia | *e1-fs/e2/e3/e4* | 00 | 000 | na |  |
| SD55 | Zernitsa | Russia | *e1-nl/e2/E3/e4* | 00 | 000 | 000 |  |
| SD56 | L315/07 | Russia | *e1-as/e2/E3/E4* | 00 | 000 | 000 |  |
| SD57 | Niva 70 | Russia | *e1-as/e2/e3/e4* | 00 | 00 | 000 |  |
| SD58 | Sibiryachka | Russia | *e1-nl/e2/E3/e4* | 00 | 000 | 000 |  |
| SD59 | Eldorado | Russia | *e1-as/e2/E3/E4* | 00 | 000 | na |  |
| SD60* | Garmonia | Russia | *e1-as/e2/E3/E4* | 00 | 00 | 00 |  |
| SD61 | Romantika | Russia | *E1/e2/E3/e4* | 00 | 00 | 00 |  |
| SD62 | Belgorodskaya 6 | Russia | *e1-as/e2/e3/e4* | 00 | 00 | 00 |  |
| SD63 | Veidelevskaya 17 | Russia | *e1-as/e2/E3/e4* | 00 | 00 | na |  |
| SD64 | Yantarnaya | Russia | *e1-as/e2/E3/E4* | 00 | 00 | na |  |
| SD65 | Altom | Russia | *e1-nl/e2/e3/e4* | 00 | 00 | 00 |  |
| SD66 | SibNIISHOZ 6 | Russia | *e1-fs/e2/E3/E4* | 00 | 000 | 000 |  |
| SD67 | PEP 26 | Russia | *e1-as/e2/e3/E4* | 00 | 000 | 000 |  |
| SD68 | Belor | Russia | *e1-nl/e2/e3/e4* | 00 | 00 | 000 |  |
| SD69 | Soer 345 | Russia | *e1-as/e2/E3/E4* | 00 | 00 | 00 |  |
| SD70 | Gribskaya Kormovaya | Russia | *e1-as/e2/E3/E4* | 00 | 00 | 00 |  |
| SD71 | Krasivaya mechta | Russia | *e1-as/e2/E3/e4* | 00 | 000 | na |  |
| SD72 | Carola | USA | *E1/e2/E3/e4* | 00 | 00 | 00 |  |
| SD73 | Daksoy | USA | *e1-as/e2/het/e4* | 0 | 00 | na |  |
| SD74 | Dawson | USA | *e1-as/e2/E3/E4* | I | 0 | 00 |  |
| SD75 | L5582 | Uzbekistan | *e1-as/e2/e3/E4* | I | 0 | 00 |  |
| SD76 | USHI 6 | Ukraine | *e1-as/e2/E3/E4* | 00 | 000 | 000 |  |
| SD77 | Prikorpat'ska 81 | Ukraine | *E1/e2/E3/e4* | 00 | 00 | 00 |  |
| SD78 | Chernovitskaya 7 | Ukraine | *E1/e2/E3/e4* | 00 | 00 | 00 |  |
| SD79 | Spritna | Ukraine | *E1/e2/E3/e4* | 0 | 00 | 00 |  |
| SD80* | Terek | Ukraine | *e1-nl/e2/E3/E4* | I | 00 | na |  |
| SD81 | Ustya | Ukraine | *e1-nl/e2/E3/e4* | 00 | 00 | na |  |
| SD82 | Horol | Ukraine | *e1-nl/e2/E3/E4* | 0 | 0 | na |  |
| SD83 | Yug 30 | Ukraine | *e1/e2/e3/e4* | 00 | 00 | 00 |  |
| SD84 | Estophyta | Ukraine | *e1-as/e2/E3/E4* | 00 | 0 | 00 |  |
| SD85 | Podyaka | Ukraine | *e1-as/e2/E3/E4* | 0 | 0 | 00 |  |
| SD86 | Victorina | Ukraine | *e1-as/e2/E3/e4* | 00 | 00 | 00 |  |
| SD87 | Annushka | Ukraine | *e1-as/E2/E3/E4* | 00 | 000 | na |  |
| SD88 | Amour | France | *e1-fs/E2/E3/E4* | II | 0 | na |  |
| SD89 | Kalmit | France | *E1/e2/e3/E4* | 0 | 000 | 00 |  |
| SD90 | Sepia | France | *e1-as/e2/E3/e4* | 0 | 00 | 00 |  |
| SD91 | Amphor | France | *e1-nl/e2/E3/e4* | I | 0 | 00 |  |
| SD92 | Toury | Chech Republic | *e1-nl/E2/e3/E4* | I | 00 | 000 |  |
| SD93 | Rana | Chech Republic | *e1-as/e2/e3/E4* | 00 | 000 | 000 |  |
| SD94 | Turijskaja masnaja | Chech Republic | *e1-as/e2/e3/E4* | 00 | 00 | 00 |  |
| SD95 | Fiskeby V | Sweden | *e1-as/e2/e3/e4* | 00 | 000 | 000 |  |
| SD96 | Fiskeby III | Sweden | *E1/e2/e3/e4* | 00 | 00 | 00 |  |
| SD97 | Oyachi 2 | Japan | *E1/E2/e3/E4* | 0 | 00 | 000 |  |
| SD98 | L6287 | Japan | *e1-as/e2/E3/E4* | 00 | 000 | 00 |  |
| SD99 | L6792 | Denmark | *e1-as/e2/e3/E4* | 00 | 000 | na |  |
| SD100 | Sito | Germany | *e1-nl/e2/e3/E4* | 00 | 000 | 000 |  |
| SD101 | Johny | na | *E1/e2/E3/e4* | 00 | 00 | na |  |
| SD102 | Semu 315 | Chech Republic | *E1/E2/e3/E4* | 00 | 000 | 000 |  |
| SD103 | 308/1 | KAZ, NK | *e1-as/e2/E3/E4* | 00 | 000 | 000 |  |
| SD104 | 422/1 | KAZ, NK | *e1-as/e2/E3/E4* | 00 | 000 | 000 |  |
| SD105 | 186/1 | KAZ, NK | *e1-as/e2/E3/E4* | 00 | 000 | 000 |  |
| SD106 | 173/1 | KAZ, NK | *e1-as/e2/e3/E4* | 00 | 000 | 000 |  |
| SD107 | 126/1 | KAZ, NK | *e1-as/e2/E3/E4* | 00 | 000 | 000 |  |
| SD108 | 209/1 | KAZ, NK | *e1-as/e2/e3/e4* | 00 | 000 | 000 |  |
| SD109 | 261/1 | KAZ, NK | *e1-as/e2/E3/E4* | 00 | 000 | 000 |  |
| SD110 | 350/1 | KAZ, NK | *e1-as/e2/E3/E4* | 00 | 000 | 000 |  |
| SD111 | 362/2 | KAZ, EK | *E1/e2/e3/E4* | I | 00 | 00 |  |
| SD112 | 371/2 | KAZ, EK | *e1-as/e2/e3/E4* | 0 | 0 | 00 |  |
| SD113 | 407/2 | KAZ, EK | *e1-fs/e2/e3/E4* | I | 00 | 00 |  |
| SD114 | 404/2 | KAZ, EK | *E1/e2/e3/E4* | I | 00 | 00 |  |
| SD115 | 370/2 | KAZ, EK | *e1-fs/e2/e3/E4* | 0 | 00 | 00 |  |
| SD116 | Zara | KAZ, SEK | *E1/e2/E3/E4* | I | na | na |  |
| SD117 | Roza | KAZ, SEK | *e1-as/e2/E3/E4* | II | na | na |  |
| SD118 | Misula | KAZ, SEK | *e1-as/e2/e3/E4* | I | na | na |  |
| SD119 | Almaty | KAZ, SEK | *e1-as/e2/E3/E4* | I | na | na |  |
| SD120 | Zhalpaksay | KAZ, SEK | *E1/e2/e3/E4* | I | na | na |  |

MG, maturity Groups; SEK15, South-east Kazakhstan 2015; EK15, East Kazakstan 2015; NK, North Kazakhstan 2015; KAZ, Kazakhstan.

* - accessions were analyzed in a high latitude region of China [7].
